# 83569 Receipt of Pharmacologic Weaning Therapy and Developmental Delay

**DOI:** 10.1017/cts.2021.738

**Published:** 2021-03-30

**Authors:** Angela G. Campbell, Pengyue Zhang, Sami Gharbi, Sarah Wiehe

**Affiliations:** Indiana University School of Medicine

## Abstract

ABSTRACT IMPACT: This study evaluates the long term effects of pharmacologic weaning therapy for opiate exposed infants. OBJECTIVES/GOALS: Infants born to chronic opioid users often suffer from neonatal abstinence syndrome (NAS), a condition characterized by tremors, diarrhea, hyperirritability and an inconsolable high-pitched cry. Symptoms are treated with pharmacologic weaning therapy, but long-term effects of this treatment have not been established. METHODS/STUDY POPULATION: A sample of infants born between 2011-2017 was obtained from a large metropolitan hospital system. All infants who were exposed to opioids and received a Finnegan score were included in the sample (N=1,807). The analysis utilizes three dependent variables to measure developmental delay: motor delay, language delay or any delay, which includes general/non-specific delay in addition to motor and language delay. The treatment is defined as receipt of pharmacologic therapy with methadone or morphine. Maximum Finnegan score was also included as a continuous measure of the extent of the infant’s withdrawal symptoms. Linear models were utilized to determine a relationship between pharmacologic therapy and developmental delay with Maximum Finnegan score as an interaction term. RESULTS/ANTICIPATED RESULTS: In the linear models examining the main effects of weaning therapy on developmental delay, there was no relationship between pharmacologic therapy and motor delay (p=.260), language delay (p=.542) or any developmental delay (p=.176). When maximum Finnegan score was entered into the model as an interaction term the relationships were not significant. DISCUSSION/SIGNIFICANCE OF FINDINGS: These results suggest that while pharmacologic weaning is an appropriate treatment for withdrawal symptoms in infants, it is not a deterrent against developmental delays associated with NAS. This provides support suggest an increased focus on non-pharmacologic interventions such as breastfeeding as the first line of treatment for NAS infants.

